# Study on Adsorption Mechanism and Failure Characteristics of CO_2_ Adsorption by Potassium-Based Adsorbents with Different Supports

**DOI:** 10.3390/ma11122424

**Published:** 2018-11-30

**Authors:** Bao-guo Fan, Li Jia, Yan-lin Wang, Rui Zhao, Xue-song Mei, Yan-yan Liu, Yan Jin

**Affiliations:** College of Electrical and Power Engineering, Taiyuan University of Technology, Taiyuan 030024, China; fanbaoguo@tsinghua.org.cn (B.-g.F.); 18734869558@163.com (L.J.); 13453122526@163.com (Y.-l.W.); 19834430509@163.com (R.Z.); explorer2018@163.com (X.-s.M.); 18334703500@163.com (Y.-y.L.)

**Keywords:** potassium-based adsorbent, load modification, CO_2_ adsorption, failure, kinetics, microscopic characteristics

## Abstract

In order to obtain the adsorption mechanism and failure characteristics of CO_2_ adsorption by potassium-based adsorbents with different supports, five types of supports (circulating fluidized bed boiler fly ash, pulverized coal boiler fly ash, activated carbon, molecular sieve, and alumina) and three kinds of adsorbents under the modified conditions of K_2_CO_3_ theoretical loading (10%, 30%, and 50%) were studied. The effect of the reaction temperature (50 °C, 60 °C, 70 °C, 80 °C, and 90 °C) and CO_2_ concentration (5%, 7.5%, 10%, 12.5%, and 15%) on the adsorption of CO_2_ by the adsorbent after loading and the effect of flue gas composition on the failure characteristics of adsorbents were obtained. At the same time, the microscopic characteristics of the adsorbents before and after loading and the reaction were studied by using a specific surface area and porosity analyzer as well as a scanning electron microscope and X-ray diffractometer. Combining its reaction and adsorption kinetics process, the mechanism of influence was explored. The results show that the optimal theoretical loading of the five adsorbents is 30% and the reaction temperature of 70 °C and the concentration of 12.5% CO_2_ are the best reaction conditions. The actual loading and CO_2_ adsorption performance of the K_2_CO_3_/AC adsorbent are the best while the K_2_CO_3_/Al_2_O_3_ adsorbent is the worst. During the carbonation reaction of the adsorbent, the cumulative pore volume plays a more important role in the adsorption process than the specific surface area. As the reaction temperature increases, the internal diffusion resistance increases remarkably. K_2_CO_3_/AC has the lowest activation energy and the carbonation reaction is the easiest to carry out. SO_2_ and HCl react with K_2_CO_3_ to produce new substances, which leads to the gradual failure of the adsorbents and K_2_CO_3_/AC has the best cycle failure performance.

## 1. Introduction

Global climate change caused by greenhouse gas emissions is a hot issue in our modern society, which is related to the development and survival of the whole mankind. China’s carbon emissions account for 29% of the global total and rank first in the world [1]. The current CO_2_ emission reduction technologies are mainly divided into four ways: pre-combustion decarbonization [2,3], chemical chain circulation [4,5], pure oxygen combustion [6,7], and post-combustion capture [8,9,10,11]. Among them, CO_2_ capture and storage (CCS) technology has been widely used [12,13,14,15], but its cost is high [16,17]. However, alkali metal carbonates such as Na_2_CO_3_ and K_2_CO_3_ have become the promising CO_2_ adsorbents due to their low cost, low secondary pollution, and high cycle efficiency [18,19].

It has been found that potassium-based adsorbents can remove CO_2_ at a low temperature (60–80 °C) under the conditions in which water vapor is adsorbed [20]. However, its adsorption efficiency is low. Some researchers have modified the potassium-based adsorbent by using activated carbon (AC), MgO, TiO_2_ [21], and a 5A molecular sieves [22] and studied the carbonation reaction by thermogravimetric analyzer. They found that the CO_2_ adsorption rate of K_2_CO_3_/AC adsorbent increased by 73% as the AC loading increased from 9% to 33% under the conditions of 60 °C and 10% CO_2_ [23]. When the Al_2_O_3_ loading increased from 12.8% to 36.8%, the CO_2_ adsorption rate of the K_2_CO_3_/Al_2_O_3_ adsorbent increased by 62% [24]. However, the above studies only use the thermogravimetric analyzer to study the carbonation reaction process by the weight loss of the adsorbent. The mass of the adsorbent involved in the reaction is small and the range of loading is narrow.

Since the potassium-based adsorbent can be regenerated in the temperature range of 120 to 200 °C, it provides the possibility of an absorption-regeneration cycle of the adsorbent, which achieves the efficient removal of CO_2_. At the same time, the material composition and microstructure of fly ash in coal-fired power plants are very similar to those of activated carbon. Hence, the use of fly ash as a support can make up for the disadvantages of low utilization efficiency of fly ash alone and also modify the potassium-based adsorbent to improve the removal efficiency of CO_2_. However, due to the large difference between the combustion mode of pulverized coal (PC) and circulating fluidized bed boiler (CFB) and the fuel used, the chemical composition and microstructure of the fly ash produced are quite different.

In addition, the reaction and adsorption kinetics studies have become an important method for predicting the adsorption rate-determining step and analyzing the adsorption mechanism. It is widely used in the adsorption of heavy metals from the liquid phase and the adsorption of SO_2_ and NO on the surface of solid adsorbents. At present, there are very limited studies on the adsorption kinetics, thermodynamics, and adsorption equilibrium of CO_2_ on the surface of adsorbents.

In summary, the adsorption of CO_2_ by potassium-based adsorbents is related to its characteristics and the focus of the above studies is scattered. Although there have been studies on the modification of potassium-based adsorbents by different supports, the related effects vary greatly depending on the type of support. Among them, the studies on the modification using fly ash as a support have rarely been reported. The study of adsorption kinetics of CO_2_ by adsorbents is also relatively small and the relevant mechanisms are not fully explained. On the basis of different supports’ effects on the CO_2_ adsorption characteristics of potassium-based adsorbents combined with the microscopic properties of the adsorbent, the mechanism of carbonation is studied using reaction and adsorption kinetics. Moreover, the effect of flue gas composition on the failure characteristics of the adsorbent is studied, which will provide a theoretical basis for future CO_2_ removal methods.

## 2. Research Object and Method

### 2.1. Preparation and Characterization of Samples

K_2_CO_3_ (Bodi chemical industry, Tianjin, China) was chosen as the active component of the adsorbent sample in this study and circulating fluidized bed fly ash (CFA) (Ping Shuo power plant, Pingshuo, China), pulverized coal furnace fly ash (PFA) (Datang Taiyuan Second Thermal Power Plant, Tai Yuan, China), activated carbon (AC) (Guang Fu Technology, Tianjin, China), 5A molecular sieve (5A) (Hua Kang, Gongyi, China), and γ-aluminum oxide (γ-Al_2_O_3_) (Heng Xing, Tianjin, China) were used as supports. The adsorbents were prepared by impregnating. The theoretical loading of K_2_CO_3_ were selected 10%, 30%, and 50%, respectively. 100 g supports were added to an aqueous solution containing a certain amount of K_2_CO_3_ (10 g, 30 g, and 50 g) in 500 mL deionized water. Then, it was stirred with a magnetic stirrer (Guang Ming, Beijing, China) for 10 h at room temperature. Thereafter, the mixture was dried in the oven (Gang Yuan, Tianjin, China) for 8 h at 105 °C. The dried samples were then calcined in a muffle furnace (Ke Jing, Zhengzhou, China) for 4 h at 300 °C. Lastly, the samples were crushed and screened to a particle size range within 75 μm by the sieve shaker (Xin Da, Shaoxing, China) to get the final adsorbent.

The actual loading represents the ratio of the active component K_2_CO_3_ loaded on the support particles in the prepared adsorbent, which is shown in Equation (1). The theoretical and corresponding actual loadings of the five supports are shown in Table 1.

(1) $$L_{R}=\frac{m_{f}-m_{i}}{m_{i}}\times 100\%$$

where L_R_ is the actual loading, %; m_f_ is the mass of the sample after loading, g; mi is the mass of the support particles, g.

In addition, the content of the K element in the adsorbent was analyzed by ARL9800XP X-ray Fluorescence (XRF) (ARL, Berne, Switzerland) and a more accurate actual load was obtained. The results are shown in Table 2. It can be seen from the table that the actual loading calculated by the XRF characterization method is similar to the calculation result of Equation (1) and the results of this paper are verified.

In order to obtain the microscopic characteristics of the prepared adsorbent, the N_2_ adsorption and desorption experiments were carried out by ASAP 2460 analyzer (Micromeritics, Norcross, GA, USA). The specific surface area was calculated by the Brunauer-Emmett-Teller (BET) equation and the pore structure parameters of the adsorbent were obtained by the Barrett-Joyner-Halenda (BJH) method. The surface morphology of the adsorbent was obtained by the Nova Nano SEM 50 scanning electron microscopy (Thermo Fisher Scientific, Hillsboro, OR, USA) and the crystal structure of the adsorbent was obtained by the X/max-2500 X-ray diffractometer (XRD) (Rigaku, Tokyo, Japan).

### 2.2. Fixed Bed CO_2_ Adsorption Experiment System

**Figure 1:**
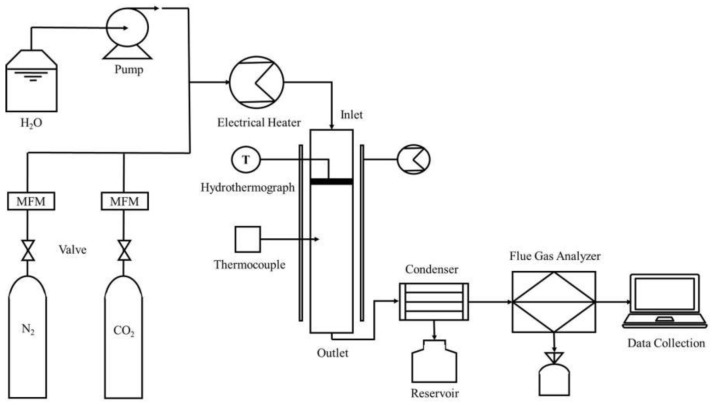
Carbonation reaction system.

The fixed bed carbon adsorption experimental system is shown in Figure 1. It is mainly composed of a simulated gas production system, a reaction system, and a data acquisition and processing system. The simulated flue gas N_2_/CO_2_/H_2_O in the experiment was provided by a gas distribution system in which the flow rate of water was controlled by a Series III metering pump (SSI, Cincinnati, OH, USA) and gasified into water vapor by electric heating. It was also thoroughly mixed with N_2_/CO_2_. During the carbonation reaction, the CO_2_ concentration was monitored online by the MOT (Monitor) series gas detector (Keernuo, Shenzhen, China).

In the experiment of carbonation of adsorbent, 5 g adsorbent was placed in the reactor with a simulated flue gas volume of 500 mL (water vapor concentration is 10%). Since the reaction temperature range of carbonation is 50 to 90 °C, considering that the actual flue gas environment of the power plant, the reaction temperatures were selected as 50 °C, 60 °C, 70 °C, 80 °C, and 90 °C while the CO_2_ concentrations were selected as 5%, 7.5%, 10%, 12.5%, and 15%, respectively. Furthermore, the CO_2_ adsorption characteristics of the adsorbent were evaluated by the CO_2_ adsorption rate η as shown in Equation (2).

(2) $$\eta =\frac{\frac{\Delta m}{2\times M_{{\mathrm{KHCO}}_{3}}}\times M_{K_{2}{\mathrm{CO}}_{3}}}{m_{k_{2}{\mathrm{CO}}_{3}}}\times 100\%.$$

where Δm is the weight gain of the sample during the reaction, g. $M_{K_{2}{\mathrm{CO}}_{3}}$ is the relative molecular mass of K_2_CO_3_, M_r_ = 138. $M_{{\mathrm{KHCO}}_{3}}$ is the relative molecular mass of HKCO_3_ and M_r_ = 100 $M_{k_{2}{\mathrm{CO}}_{3}}$ is the mass of the adsorbent before the reaction, g.

Moreover, in order to obtain the failure characteristics of the adsorbents under different flue gas composition, the adsorbents sample adsorbed with CO_2_ were filled into the fixed bed carbon adsorption experiment system as described above to perform multiple adsorption/regeneration cycle experiments. During the cycle experiment, NO (0.05%), SO_2_ (0.05%), and HCl (0.05%) were added to the atmosphere of the carbonation reaction. Regeneration experiments were carried out in an N_2_ atmosphere and the reaction temperature was 200 °C.

## 3. Results and Discussion

### 3.1. Effect of Loading and Adsorption Conditions on Carbonation Reaction

#### 3.1.1. Carbonation Reaction Characteristics of Adsorbents under Different Load Conditions

**Figure 2:**
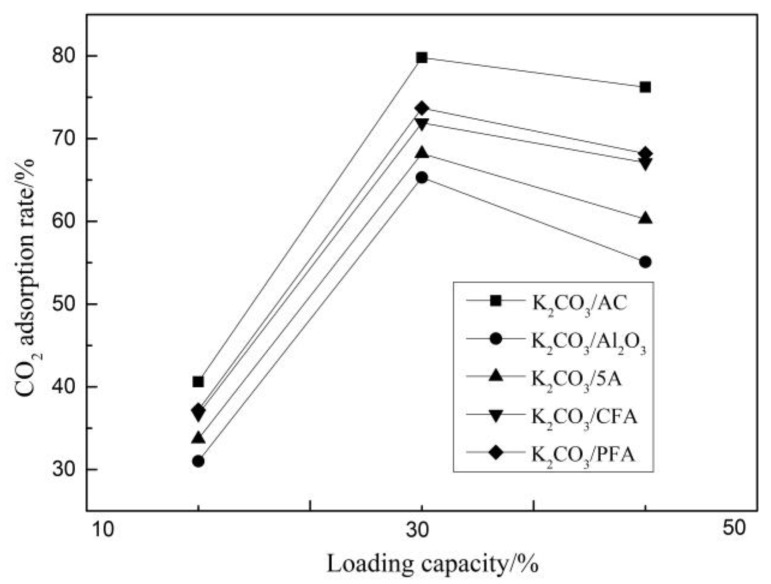
CO_2_ adsorption rate at different loading capacities.

In order to obtain the optimal loading of adsorbents of different support types, the carbonation reaction of the modified potassium-based adsorbents were carried out at 12.5% CO_2_ and 70 °C. The results are shown in Figure 2.

Among all the adsorbents, the K_2_CO_3_/AC sample has the highest CO_2_ adsorption rate under different loading conditions and the actual loading of K_2_CO_3_ in this adsorbent is also the largest. Among the different adsorbents, when the theoretical loading of K_2_CO_3_ is 10%, the adsorption rate of CO_2_ is in the lower range of 30% to 40%. The CO_2_ adsorption rates of the five adsorbents increased significantly as the theoretical loading increased to 30%, which reached 79.8%, 65.3%, 68.2%, 71.9%, and 73.7%, respectively. However, when the theoretical loading reaches 50%, the CO_2_ adsorption rate shows a slight downward trend. This is because the loading of the active component on the surface of the support is mainly divided into three stages: unsaturated load, saturated load, and multilayer load. With the increase in K_2_CO_3_ loading from 10% to 30%, the load of the active component undergoes a process of an unsaturated state to saturation. When the loading increases from 30% to 50%, the load state is switched from a saturated load to a multi-layer load and, at this time, the active component has an overlapping multi-layer load phenomenon on the surface of the support. Hence, when the adsorbent undergoes a carbonation reaction, the outermost active component will first undergo a carbonation reaction to form KHCO_3_. While KHCO_3_ is a dense and smooth material and the formed product layer hinders the reaction of CO_2_ with the active components of the inner layer, it results in a slight decrease in the adsorption rate of CO_2_.

#### 3.1.2. Carbonation Reaction Characteristics of Adsorbents under Different Adsorption Conditions

**Figure 3:**
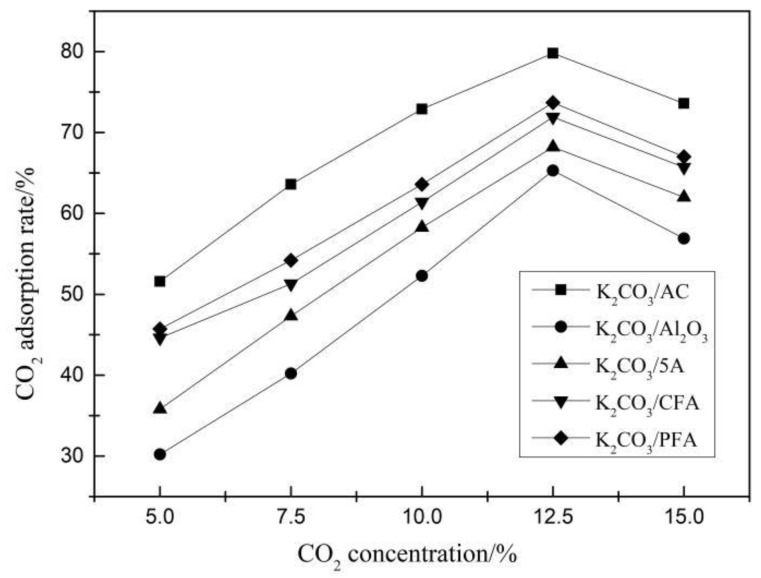
CO_2_ adsorption rate at different CO_2_ concentrations.

**Figure 4:**
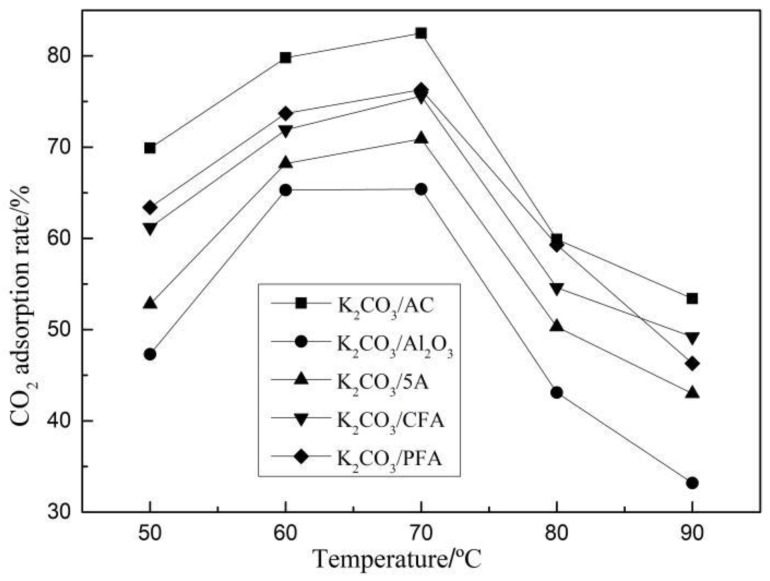
CO_2_ adsorption rate at different temperatures.

Carbonation reaction experiments of five kinds of adsorbents with the optimal theoretical loading (30%) were carried out under different CO_2_ concentrations and reaction temperatures. When studying the effect of different CO_2_ concentrations on the adsorption rate, the temperature was set to 70 °C. When studying the effect of different temperatures on the adsorption rate, the CO_2_ concentration was set to 12.5% and the results are shown in Figure 3 and Figure 4.

It can be concluded from Figure 3 that the CO_2_ adsorption rate increases first and then decreases with the increase of CO_2_ concentration and the optimal CO_2_ concentration is 12.5%. This is because the difference between the CO_2_ concentration on the surface of the adsorbent and the CO_2_ concentration of the atmosphere is a driving force during the entire adsorption process. Hence, increasing the initial concentration of CO_2_ can accelerate the adsorption rate, which promotes the CO_2_ adsorption performance of the adsorbent. However, when the number and activity of the adsorbent sites on the adsorbents surface are certain, the increase of the concentration of CO_2_ will also increase the adsorption capacity of the adsorbents, which leads to the decrease of the adsorption efficiency and the rate of the adsorbents. Therefore, the CO_2_ adsorption performance of the adsorbent is inhibited. Among all the adsorbents, the CO_2_ adsorption rate of K_2_CO_3_/AC is the largest and the degree of carbonation reaction of K_2_CO_3_/Al_2_O_3_ and K_2_CO_3_/5A is relatively close while the effect is poor.

It can be seen from Figure 4 that the optimal reaction temperature is 70 °C and the adsorption rates at 60 °C and 70 °C are relatively close. The CO_2_ adsorption rate decreases significantly with the increase of temperature at the range of 70 to 90 °C. The adsorption of CO_2_ on the supporting potassium-based adsorbent existed in both physical and chemical adsorption and the chemisorption was dominant. With the increase of the reaction temperature, the diffusion process of CO_2_ in the surface of the adsorbent and the internal pores is accelerated, which is beneficial to the reaction of CO_2_ with the adsorbent. Meanwhile higher temperature leads to easier breakage of chemical bonds and the lower energy barrier of the active component, which promotes chemical reaction. However, the reaction of K_2_CO_3_, CO_2_, and H_2_O to form KHCO_3_ in the range of 120 to 200 °C is reversible. Hence, the activity of K_2_CO_3_ decreases and the reaction process tends to reverse when the temperature rises from 70 °C to 90 °C, which leads to the rapid decrease of the CO_2_ adsorption rate.

In conclusion, the optimal theoretical loading of the adsorbent is 30% and the optimal adsorption reaction conditions include a CO_2_ concentration of 12.5% and a reaction temperature of 70 °C. Moreover, among the active adsorbents prepared by five different supports, the K_2_CO_3_/AC adsorbent has the highest degree of carbonation reaction, which is followed by K_2_CO_3_/CFA, K_2_CO_3_/PFA, and K_2_CO_3_/5A adsorbents. K_2_CO_3_/Al_2_O_3_ has the worst activity.

### 3.2. Microscopic Characteristics

In this paper, the microscopic properties (pore structure and surface morphology) of the adsorbent before and after modification at the optimal theoretical loading were studied. Meanwhile, the composition and crystal structure of the adsorbent before and after carbonation reaction were studied.

#### 3.2.1. Pore Structure

The pore structure parameters affecting the adsorption characteristics of the adsorbent mainly include specific surface area, specific pore volume, and pore size distribution. By taking the adsorbent before and after modification under optimal theoretical loading as the research object. N_2_ adsorption/desorption experiment was carried out at a low temperature to study the pore structure of the adsorbent. The results are shown in Table 3. When studying the effect of the pore structure on the ability of the adsorbent to adsorb CO_2_, the specific surface area per unit volume *Z* is introduced to characterize its pore richness [25,26], which is represented by Formula (3) below.

(3) Z=S0V0

where *S*_0_ is the BET specific surface area of the adsorbent, m^2^/g. *V*_0_ is the sum of the specific pore volumes of the adsorbent, cm^3^/g.

Furthermore, surface fractal dimension of the adsorbent can be selected as one of the evaluating parameter to characterize its pore structure. When the fractal dimension is 2, the surface of the object is smooth and regular. When the fractal dimension is close to 3, the surface structure becomes disordered and disordered. Its value can be obtained by the FHH (Frenkel, Halsey and Hill) equation [27]. Preifer et al. [28] believe that the FHH theory applies to the adsorption and desorption processes in cryptopores (one to several ten nm). The fractal FHH equation is shown in Equation (4) and the fractal dimension of the internal pore surface of the particle can be determined by the N_2_ adsorption isotherm. In the adsorption process, the adsorption interface is mainly affected by the Van der Waals Force in which the relationship between the constant *S_N_* and the fractal dimension *D_S_* is shown in Equation (5). Thus, the fractal dimension can be calculated by simultaneous Equations (4) and (5).

(4) $$\frac{V}{V_{m}}=k\cdot {\left(ln\frac{P_{0}}{P}\right)}^{S_{N}}$$

(5) $$S_{N}=D_{S}-3$$

where *V_m_* is the single layer saturated adsorption capacity, *k* is a constant, *P* is the pressure of the adsorbate, *P*_0_ is the saturated vapor pressure of the adsorbate, *S_N_* is a constant related to the adsorption mechanism and fractal dimension *D*, and *D_S_* is the fractal dimension.

From Table 3, the microstructure of K_2_CO_3_ is poor and its specific surface area and the cumulative pore volume are small. The adsorption of CO_2_ by the adsorbent mainly depends on the chemical reaction. In addition, in all the adsorbents after loading, the BET specific surface area and the cumulative pore volume decreased greatly and the pore richness Z also showed a decreasing trend, which indicates that a large amount of K_2_CO_3_ adhered to the surface and pores of the support after loading.

Since the surface area and cumulative pore volume of AC are much larger than other supports, the active component is most loaded, which is beneficial to the carbonation reaction of the adsorbent after the loading modification. The specific surface area of the two kinds of fly ash decreased from 80.85 m^2^/g and 99.70 m^2^/g to 47.02 m^2^/g and 73.17 m^2^/g before and after loading and is only lower than AC and K_2_CO_3_/AC. However, the cumulative pore volume of both before and after loading is small. When the 5A molecular sieve was used as the support, the specific surface area and cumulative pore volume of the adsorbent before and after loading decreased most clearly, which decreased by 80.94% and 67.88%, respectively. This is because, during the load process, the inside of the molecular sieve is plastically deformed due to surface tension and a large number of active components fill the pores, which results in deterioration of the microstructure. In addition, Al_2_O_3_ has a smooth and dense structure. Even though the pore structure is improved after loading K_2_CO_3_, the lifting effect is not significant.

Combined with the results of CO_2_ adsorption, it can be concluded that the type of support and the BET specific surface area of the adsorbent after loading modification determine the degree of the carbonation reaction while the cumulative pore volume and pore size distribution have less influence. This is because the process of adsorbing CO_2_ by the active component K_2_CO_3_ on the support is a chemical reaction, which is dominant throughout the adsorption process. The larger the surface area, the more active sites on the surface of the support can adhere to K_2_CO_3_, which increases the adsorption of CO_2_ by the adsorbent. When the cumulative pore volume is large, a large number of active components are attached to the pores. However, KHCO_3_ formed by the reaction of external K_2_CO_3_ and CO_2_ has a dense and less porous substance, which will hinder the diffusion of CO_2_ into the pores and inhibit the continuous carbonation reaction. At the same time, it can be concluded that the fractal dimension and the CO_2_ adsorption rate also have a proportional relationship and, with the increase of the fractal dimension, the surface morphology of the adsorbent gradually becomes irregular, which is beneficial for the sufficient contact of the active component with CO_2_. This enhances the adsorption of CO_2_ by the adsorbent.

#### 3.2.2. Surface Morphology

**Figure 5:**
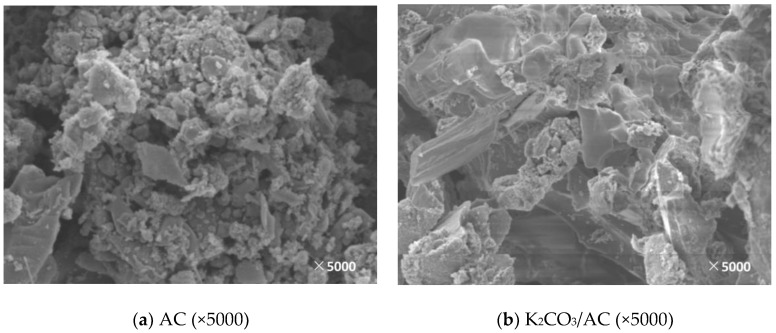
SEM image before and after AC loading. (**a**): The SEM image of AC; (**b**): The SEM image of K_2_CO_3_/AC.

**Figure 6:**
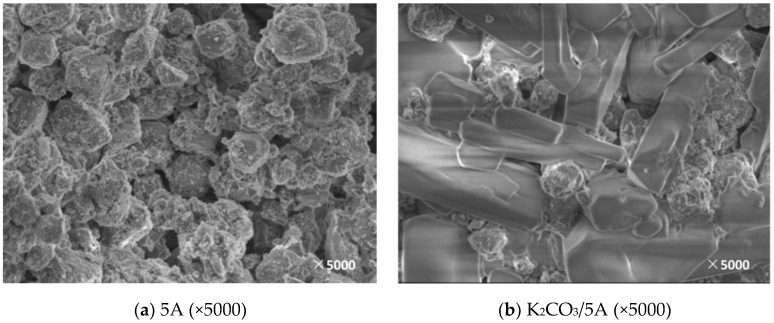
SEM image before and after 5A loading. (**a**): The SEM image of 5A; (**b**): The SEM image of K_2_CO_3_/5A.

**Figure 7:**
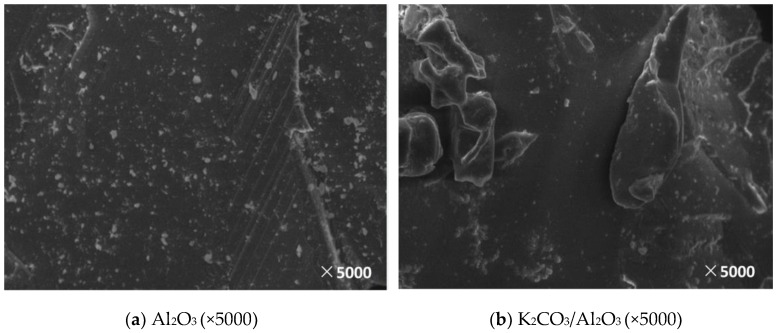
SEM image before and after Al_2_O_3_ loading. (**a**): The SEM image of Al_2_O_3_; (**b**): The SEM image of K_2_CO_3_/Al_2_O_3_.

**Figure 8:**
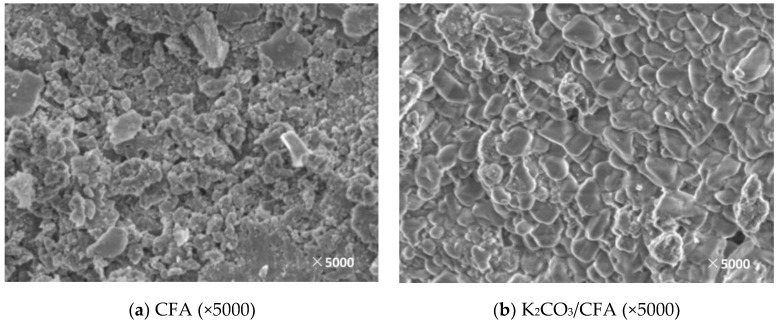
SEM image before and after CFA loading. (**a**): The SEM image of CFA; (**b**): The SEM image of K_2_CO_3_/CFA.

**Figure 9:**
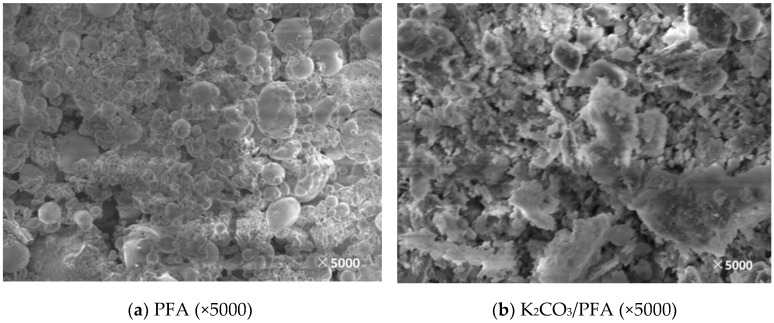
SEM image before and after PFA loading. (**a**): The SEM image of PFA; (**b**): The SEM image of K_2_CO_3_/PFA.

In this paper, the support and corresponding modified adsorbent samples were observed by SEM and the surface morphology and microstructure of the supports and the adsorbents obtained under different modification conditions were obtained, which is shown in Figure 5, Figure 6, Figure 7, Figure 8 and Figure 9.

As can be seen from Figure 5, the surface of the support AC has a rough surface and an irregular block structure. After the active component of K_2_CO_3_ is loaded, the original block structure is broken by the impregnation process and becomes a large number of irregular small particles. The surface and pores of the formed adsorbent are filled with a large amount of active components and the roughness is intensified. However, there are still significant gaps between the particles, which means the overall arrangement is loose.

Figure 6 shows the SEM results before and after loading the 5A molecular sieve. The molecular sieve particles before loading have a spherical structure with a rough surface and an abundant pore structure. The pores of the adsorbent after the loading are filled with a large amount of K_2_CO_3_, which confirmed the results of the previously mentioned reduction in a specific surface area and a cumulative pore volume. At the same time, it can be concluded that K_2_CO_3_ is loaded in a multi-layered manner in the pores. Therefore, after the surface-active component is completely reacted, the resistance of CO_2_ into the internal channel increases and the carbonation reaction is hindered.

Figure 7 shows an SEM image before and after loading Al_2_O_3_. Compared with other kinds of adsorbents, Al_2_O_3_ has a dense surface and a relatively regular layered structure. Although the surface structure is slightly improved after the impregnation, it is not significant and only a small amount of the active component is loaded on the surface.

Figure 8 shows an SEM image before and after the CFA loading. The surface morphology of the fly ash before loading is relatively rough and it is mainly composed of a dense porous structure. After loading, a large amount of the active components are deposited on the surface and the CO_2_ can be sufficiently contacted to facilitate the carbonation reaction.

Figure 9 shows the SEM image before and after the PFA loading. The surface of the fly ash before loading contains a large number of round particles. The fly ash particles are fine and the shape is relatively simple. The particle diameter of the particles is different, the maximum is not more than 10 μm, and the diameter of most particles is 1–4 μm [29]. These particles are most likely aluminosilicate or fly ash balls. After the impregnation, the surface morphology changed greatly, the spherical particles disappeared, and the surface was loaded by the active components. However, the overall pore structure did not change significantly.

#### 3.2.3. Lattice Structure

**Figure 10:**
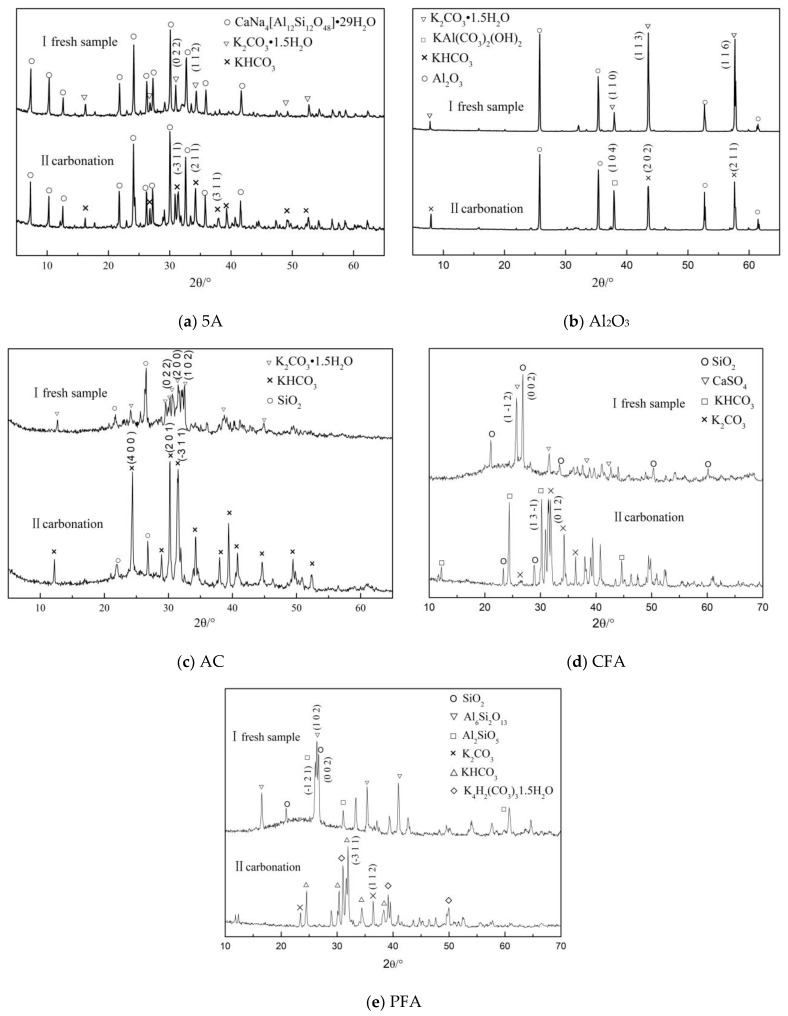
XRD diffraction pattern before and after carbonation of different adsorbents. (**a**): The XRD result of 5A; (**b**): The XRD result of Al_2_O_3_; (**c**): The XRD result of AC; (**d**): The XRD result of CFA; (**e**): The XRD result of PFA.

In order to obtain the changes of the composition of the K_2_CO_3_ adsorbent under different carbonation reactions, XRD analysis was performed on the samples before and after adsorption, which is shown in Figure 10. Among them, only the K_2_CO_3_/Al_2_O_3_ adsorbent produces a new chemical reaction in the carbonation reaction process and KAl(CO_3_)_2_(OH)_2_ is formed. The presence of Al_2_O_3_ will compete with K_2_CO_3_ for adsorption. The chemical adsorption of CO_2_ by the active component is reduced. The miller index corresponding to the strong diffraction peaks of the material K_2_CO_3_·1.5H_2_O before the reaction are (1 1 0), (1 1 3), and (1 1 6), respectively. The miller index corresponding to the three strong peaks of the main product KHCO_3_ after the reaction are (1 0 4), (2 0 2), and (2 1 1), respectively. The miller index of the K_2_CO_3_/5A and K_2_CO_3_/AC adsorbents corresponding to the strong diffraction peaks of K_2_CO_3_·1.5H_2_O before the carbonation reaction are (0 2 2), (1 1 2), and (0 2 2), (2 0 0), (1 0 2) respectively. In addition, the miller index corresponding to the strong diffraction characteristic peaks of the main product KHCO_3_ after the reaction are (1 0 4), (2 0 2), (2 1 1), and (4 0 0), (2 0 1), (−3 1 1). The main components before the CFA reaction are SiO_2_ and CaSO_4_ and the miller index corresponding to the strong diffraction characteristic peaks are (0 0 2) and (1 −1 2), respectively. The main components before the PFA reaction are SiO_2_, Al_6_Si_2_O_13_, and Al_2_SiO_5_ and the crystal miller index corresponding to the strong diffraction characteristic peaks are (0 0 2), (1 0 2), and (−1 2 1), respectively. The main products of the adsorbents with CFA and PFA as supports are KHCO_3_ and unreacted K_2_CO_3_ and the corresponding miller index are (1 3 −1), (0 1 2), and (−3 1 1), (1 1 2), respectively.

### 3.3. Reaction and Adsorption Kinetics

The intrinsic reaction of the carbonation reaction of potassium-based adsorbent is the chemical reaction of K_2_CO_3_ particles with CO_2_ and H_2_O. The chemical reaction process of K_2_CO_3_ absorbing CO_2_ is shown in Formula (6). It is a typical gas-solid non-catalytic reaction and the K_2_CO_3_ particles used in the experiment have a very small specific pore volume and are compact particles. Hence, the shrinking core model was used to describe its carbonation reaction mechanism [30].

(6) $$K_{2}CO_{3}\left(s\right)+CO_{2}\left(g\right)+H_{2}O\left(g\right)=2KHCO_{3}\left(s\right)$$

The carbonation reaction of adsorbent mainly includes three basic processes: gas film diffusion, surface adsorption, and intraparticle diffusion. Due to the rapid formation of the product layer with a larger specific volume than K_2_CO_3_ in the reaction process, the diffusion resistance of the mixed gas at the product layer is much greater than the diffusion resistance of the gas film. Hence, in the research process, the film diffusion process was ignored. Chemical reaction kinetic models and product layer diffusion kinetic models were used to study the reaction mechanism and main control forms. The chemical reaction control process is as shown in Formula (7) and the product layer diffusion control process is shown in Formula (8).

(7) $$t=\frac{\rho_{p}R_{P}}{k_{s}C_{A}^{0}}\left[1-{\left(1-\eta_{C}\right)}^{1/3}\right]$$

where k_s_ is the surface reaction control coefficient, min^−1^.

(8) $$t=\frac{\rho_{p}R_{P}^{2}}{6D_{e}C_{A}^{0}}\left[1-3{\left(1-\eta_{C}\right)}^{2/3}+2\left(1-\eta_{C}\right)\right]$$

where t is the reaction time and s. ρ_p_ is the molar density of the absorbent, mol/m^3^. R_P_ is the initial radius of the particle and m. D_e_ is the diffusion coefficient of the gas in the product layer, m^2^/s. $C_{A}^{0}$ is concentration of the reaction gas at time t = 0, mol/m^3^. n is the particle conversion rate, %.

**Figure 11:**
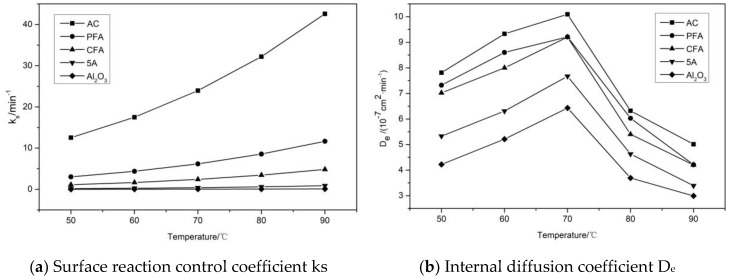
Kinetic parameters of five adsorbents at different temperatures. (**a**): Surface reaction control coefficient ks; (**b**): Internal diffusion coefficient D_e_.

In this paper, the shrinking core model was used to fit the carbonation reaction results of five adsorbents under different loading conditions at different reaction temperatures. The results are shown in Figure 11. The error between the relevant parameters obtained by fitting the equation and the experimental values is represented by the correlation coefficient R^2^. The larger the value, the closer the description of the adsorption process is to the selected model. The correlation coefficients of all adsorbent samples obtained by fitting are close to 0.99. It can be concluded that the adsorption process of CO_2_ on the adsorbent samples at different reaction temperatures is consistent with the nucleation kinetic model.

From Figure 11, it is concluded that, as the reaction temperature increases, the surface reaction rate constant k_s_ of the five adsorbents gradually increase. This is because the surface chemical reaction time is prolonged due to an increase in the reaction temperature and the carbonation pellet conversion rate is continuously increased. At the same time, the degree of reaction presents two stages. When the reaction temperature is lower than 70 °C, the diffusion coefficient D_e_ of the product layer gradually increases. This is because, during the adsorption process, the difference between the concentration of CO_2_ and H_2_O in the adsorbent surface and the adsorption atmosphere is the driving force in the whole adsorption process and the increase in temperature increases the adsorption rate and promotes the reaction, which accelerates the diffusion process of the reaction gas. In addition, when the reaction temperature is 70 °C, the carbonation conversion rate is increased to the maximum value. When the reaction temperature is higher than 70 °C, D_e_ shows a decreasing trend. This is because, as the reaction proceeds, the nuclear radius of the unreacted particles of the adsorbent gradually decreases. In addition, since the active material in the adsorbent generates more products during the early carbonation reaction, the formed KHCO_3_ product layer is more likely to wrap the surface of the adsorbent particles, which hinders the outward diffusion of the reactants in the adsorbent. At the same time, the diffusion of CO_2_ and H_2_O to the surface of unreacted particles is hindered and the internal diffusion resistance is remarkably enhanced.

In addition, since the carbonation reaction is jointly controlled by diffusion and a chemical reaction, the main control form of the reaction model is determined by the ratio ρ(ρ = (1/A_2_)/(1/A_1_) = ($\frac{\rho_{p}R_{P}}{k_{s}C_{A}^{0}}$)/($\frac{\rho_{p}R_{P}^{2}}{6D_{e}C_{A}^{0}}$)=$\frac{6D_{e}}{k_{s}R_{P}}$) of the obtained chemical reaction rate constant A_1_ and the diffusion rate constant A_2_. When ρ << 1, the reaction is mainly controlled by the intrinsic chemical reaction. When ρ >> 10, the external mass diffusion is ignored and the reaction is controlled by the diffusion process through the product layer. When ρ is between 1 and 10, the reaction is not only controlled by the intrinsic chemical reaction but also by the diffusion process through the product layer. The reaction is jointly controlled by both [31]. When the reaction temperatures are 50 °C, 60 °C, and 70 °C, respectively, the ρ values are 0.3569, 0.4623, and 0.8651, respectively, which indicates that the reaction is mainly controlled by the surface chemical reaction when the reaction temperature is lower than 70 °C. When the temperatures are 80 °C and 90 °C, the ρ values are 1.2963 and 1.3387, respectively, and the reaction is controlled by two processes, which verifies the above results.

**Figure 12:**
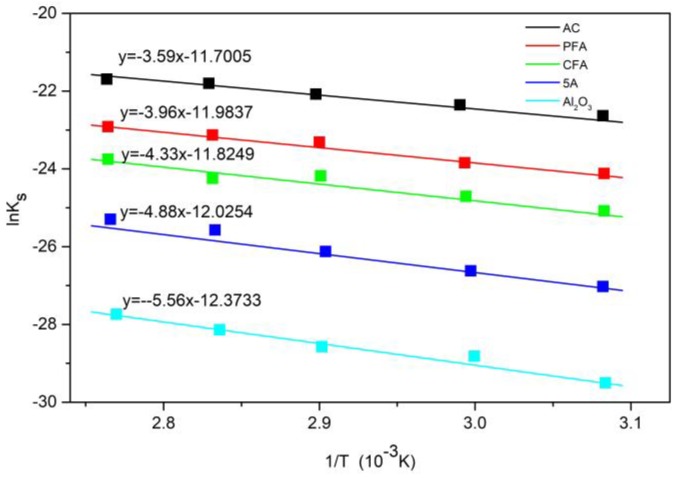
Arrhenius relational fitting diagram for a chemical reaction control phase.

In addition, in order to study the effect of temperature on the carbonation reaction process of the adsorbent, the Arrhenius equation was used. In addition, in the process of studying the influence of temperature on the carbonation reaction of adsorbent, the activation energy was obtained by the Arrhenius equation, which is shown in Formula (9). During the calculation, the logarithm of the two sides of the Arrhenius equation is obtained to obtain Equation (10). By using 1/T as the abscissa and ln(ks) as the ordinate for linear fitting, the adsorption activation energy during the adsorption reaction can be obtained. The corresponding fitting results are shown in Figure 12.

(9) $$k_{s}=A_{r1}\exp\left(-E_{a1}/\mathrm{RT}\right)$$

where T is the reaction temperature, K. A_r1_ is the pre-exponential factor of the surface reaction process, min^−1^. E_a1_ is the activation energy of the surface reaction process, kJ/mol. R is the gas constant, 8.314 J/(mol·K).

(10) $$\ln\left(k_{s}\right)=-E_{a1}/\mathrm{RT}+C$$

where C is a constant.

The activation energies of K_2_CO_3_/AC, K_2_CO_3_/PFA, K_2_CO_3_/CFA, K_2_CO_3_/5A, and K_2_CO_3_/Al_2_O_3_ are 29.82 kJ/mol, 32.91 kJ/mol, 36.02 kJ/mol, 40.57 kJ/mol, and 46.21 kJ/mol, respectively. Activation energy refers to the energy required for a molecule to change from a normal state to an active state in which a chemical reaction is likely to occur, which reflects the difficult degree of a chemical reaction. Among the five adsorbents, K_2_CO_3_/AC has the lowest activation energy and the carbonation reaction is the easiest to carry out, so the reaction rate and carbonation degree are the largest. Similarly, the activation energy of the K_2_CO_3_/Al_2_O_3_ adsorbent is the highest and the carbonation reaction is difficult, which results in the lowest degree of carbonation of the adsorbent.

It can be seen from the above information that the adsorption process of CO_2_ by adsorbents mainly includes three basic processes: external mass transfer, surface adsorption, and intraparticle diffusion. Hence, the pseudo-first order kinetic model, the pseudo-second order kinetic model, the intra-particle diffusion model, and the Elovich model are used to study the decarburization mechanism of different kinds of adsorbents and determine the rate-determining step in the adsorption process. The pseudo-first order kinetic model and the intra-particle diffusion model mainly study the physical adsorption process while the pseudo-second order kinetic model and the Elovich model are mainly used to study chemisorption. Among them, pseudo-first order kinetics mainly studies the external mass transfer process, which is shown in Formula (11). The pseudo-second order kinetic model is based on the Langmuir adsorption isotherm equation to study the formation of chemical bonds to verify that the adsorption process is dominated by chemisorption, which is shown in Formula (12). The intra-particle diffusion model is derived from the mass balance equation, which mainly studies the internal diffusion process of the pores during solid adsorption, as shown in Equation (13). The Elovich model is based on the Temkin adsorption isotherm equation and mainly describes the chemisorption process. It is similar to the pseudo-second order and the fitting results of the two models can be used to verify the accuracy of each other, which is shown in Equation (14).

(11) $$q=q_{e}\left(1-e^{-tk_{1}}\right)$$

where *q* is the adsorption amount of the adsorbent per unit mass at time *t*, g/g. *q_e_* is the adsorption amount of the adsorbent per unit mass in equilibrium, g/g. *t* is the adsorption time, min. *k*_1_ is the pseudo-first order rate constant, min^−1^.

(12) $$q=\left(q_{e}^{2}k_{2}t\right)/\left(1+q_{e}k_{2}t\right)$$

where *k*_2_ is the pseudo-second order rate constant, ng/(g·min).

(13) $${q=k}_{id}t^{1/2}+C$$

where *k_id_* is the intra-particle diffusion rate constant, g/(g·min^1/2^). *C* is the constant related to the thickness of the boundary layer, g/g. It decreases with the increase of heterogeneity and hydrophilic groups of the adsorbent surface. The larger the value, the greater the influence of the boundary layer on the adsorption.

(14) $$q=(1/\beta )ln\left({t+t}_{0}\right)-(1/\beta ){ln(t}_{0})$$

where α is the initial adsorption rate, g/(g·min^1/2^). β is a constant related to the surface coverage and activation energy, ng/g. *t*_0_ = 1/(α·β).

The four adsorption kinetic models were used to calculate and fit the adsorbent carbon adsorption experimental data. The results are shown in Table 4. The correlation coefficients R_2_ of different adsorbents are all close to 0.99. It can be concluded that the adsorption process of CO_2_ by different kinds of adsorbents is consistent with these four kinetic models. The adsorption process is affected by both physical adsorption and chemical adsorption. The adsorption of CO_2_ is not a single monolayer layer adsorption, but it is related to the adsorption site of the adsorbent. Moreover, through the predicted equilibrium adsorption amount *q_e_* in the pseudo-first order and pseudo-second order kinetic models, it can be concluded that the adsorption process of CO_2_ by the five adsorbents do not reach a saturation state within 60 min and *q_e_* has a positive correlation with its actual adsorption amount, which verifies the correctness of the fitting results. The fitting coefficient of the pseudo-first order kinetic model of K_2_CO_3_/Al_2_O_3_-30% sample is higher than that of the pseudo-second order kinetic model, which indicates that the rate-determining step is mainly a physical adsorption process, but its pseudo-first order and pseudo-second order rate constants are low, as mentioned above. This is mainly due to the poor surface pore structure and the low content of active substances. In addition, the rate-determining step of the other adsorbents is mainly chemical adsorption.

**Figure 13:**
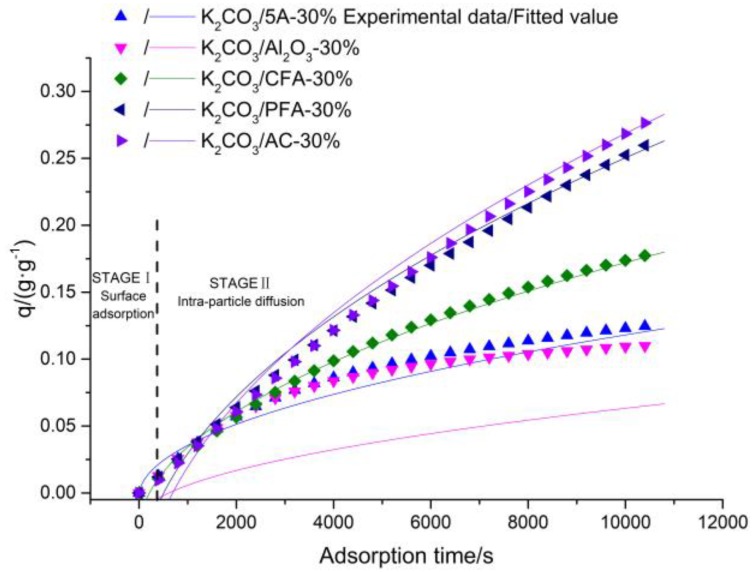
Fitting of intra-particle diffusion kinetic equation.

In addition, the intra-particle diffusion model was used to fit the cumulative CO_2_ adsorption amount per unit mass of different adsorbents and the results are shown in Figure 13. With the increase of adsorption time, the overall trend of k_id_ is increasing and the actual adsorption rate of CO_2_ decreases with the increase of adsorption time, the contradiction between the CO_2_ adsorption rate, and the internal diffusion rate indicates that there is surface adsorption during CO_2_ adsorption. Hence, the CO_2_ adsorption process can be divided into two stages: surface adsorption stage and internal diffusion adsorption stage. In the initial adsorption stage, the surface adsorption is the main form of adsorption because a large number of adsorption active sites exist on the surface of the adsorbent. Therefore, the surface adsorption rate is faster while the internal diffusion rate is smaller, which indicates that, in this stage, intra-particle diffusion does not play a leading role. When the active sites of the surface are occupied, the second stage of adsorption is carried out and diffusion adsorption occurs in the pore. At this time, the micropores and mesopores provide the adsorption active sites of CO_2_. Therefore, the adsorption rate is continuously decreased and the internal diffusion rate is increased. Moreover, the fitting curves of all intra-particle diffusion models have not passed through the origin, which is quite different from the experimental results. This indicates that the internal diffusion model cannot describe the adsorption process of CO_2_ on the adsorbent surface. The internal diffusion process is not the rate-determining step. The correlation coefficients obtained by the internal diffusion model fitting are small and significantly lower than the correlation coefficients obtained by fitting the pseudo-first order kinetic model, which indicates that the external mass transfer process is the rate-determining step for the adsorption of CO_2_ on the surface of the adsorbent relative to the internal diffusion process. In addition, although the fitting curves of the pseudo-second order model can be well matched with the experimental results, the correlation coefficients is slightly lower than the correlation coefficients obtained by the pseudo-first order model. In addition, the equilibrium CO_2_ adsorption amount obtained by fitting the pseudo-first order kinetic model is closer to the experimental results. Hence, the conclusion that the external mass transfer is the rate-determining step of CO_2_ adsorption on the adsorbent surface is further verified and the adsorption of CO_2_ at the active sites also plays a more important role. In addition, the fitting curves of the Elovich dynamic model are also in good agreement with the experimental results, which verifies the existence of the chemisorption process at the active site. The Elovich equation is based on the Temkin adsorption isotherm equation. Hence, it can be considered that the adsorption of CO_2_ on the adsorbent surface also follows the Temkin adsorption isotherm equation.

### 3.4. Study on the Effect of Flue Gas Composition on the Failure Characteristics of Adsorbents

In this paper, the adsorption/regeneration cycles of K_2_CO_3_/AC, K_2_CO_3_/Al_2_O_3_, K_2_CO_3_/5A, K_2_CO_3_/CFA, and K_2_CO_3_/PFA adsorbents were carried out and the degree of failure was characterized by the failure rate $\eta_{F}$, which is shown in Formula (15). In addition, in practical application research, it is found that when η_F_ > 20%, it indicates that the adsorbent has insufficient ability to capture CO_2_. Hence, in the study of failure characteristics in this paper, 20% is selected as the critical value of adsorbent failure.

(15) $$\eta_{F}=\left(1-\frac{\eta_{n}}{\eta_{0}}\right)\times 100\%$$

In the formula, η_n_ is the adsorption rate of CO_2_ after n times of cycle, %. η_0_ is the adsorption rate of CO_2_ in the first carbonation reaction, %.

**Figure 14:**
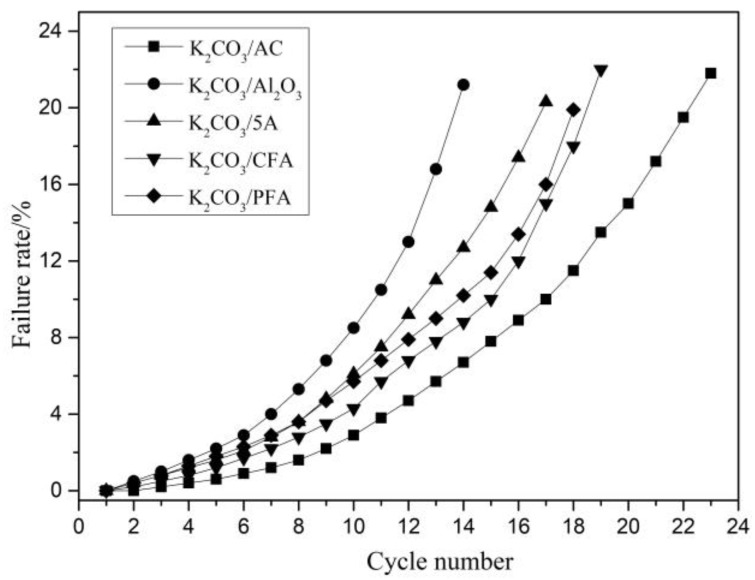
Cyclic experimental results of different adsorbents.

Figure 14 shows the variation of the failure rate of different adsorbents with the number of cycles. It can be concluded that the failure characteristics of the five adsorbents are basically the same. The failure rate of all adsorbents in the first 10 cycles increases slowly, but, as the number of cycle increases, the failure rate increases. The K_2_CO_3_/AC adsorbent has the highest number of cycles when the failure rate reaches more than 20%. The failure rate was only 21.8% until the 23rd cycle. In contrast, K_2_CO_3_/Al_2_O_3_ has the worst cycle failure characteristic and the failure rate after 14 cycles is as high as 21.2%.

In addition, in the study of the influence of flue gas composition on the failure characteristics of adsorbents, it is found that, in the presence of SO_2_, K_2_CO_3_ and SO_2_ will react similar to that in Formula (16) and Formula (17). HCl also reacts with K_2_CO_3_ to render the adsorbent failure, as shown in Formula (18). NO is stable at low temperatures and does not participate in the reaction with the active components. The five types of supports cannot react with the three acid gases under low temperature and normal pressure. Therefore, the main factors causing the difference in the failure rate are the adsorbent loading and its own CO_2_ adsorption rate.

(16) $$K_{2}CO_{3}+SO_{2}\to K_{2}SO_{3}+CO_{2}$$

(17) $$3K_{2}CO_{3}+2.5H_{2}O+SO_{2}\to K_{4}H_{2}{\left(CO_{3}\right)}_{3}\cdot 1.5H_{2}O+K_{2}SO_{3}$$

(18) $$K_{2}CO_{3}+2HCl\to 2KCl+H_{2}CO_{3}$$

## 4. Conclusions

(1) The actual loading of K_2_CO_3_/AC adsorbent is the largest and the adsorption performance of CO_2_ is the best, which is followed by the K_2_CO_3_/PFA, the K_2_CO_3_/CFA, and the K_2_CO_3_/5A adsorbent and the load modification of K_2_CO_3_/Al_2_O_3_ adsorbent is poor while the CO_2_ adsorption rate is the lowest.

(2) As the theoretical loading of the five adsorbents increases from 10% to 50%, the CO_2_ adsorption rate first increases and then decreases. The best theoretical loading is 30% and the reaction temperature of 70 °C and the concentration of 12.5% CO_2_ are the best reaction conditions.

(3) The microstructure of the adsorbents are different after modification of different supports and the cumulative pore volume plays a more important role in the adsorption process than the specific surface area. Among them, K_2_CO_3_ reacts with Al_2_O_3_ to produce KAl(CO_3_)_2_(OH)_2_ and the support and active component compete for adsorption, which reduces the chemisorption of CO_2_.

(4) With the increase of the reaction temperature, the internal diffusion resistance is significantly enhanced. K_2_CO_3_/AC has the lowest activation energy and the carbonation reaction is the easiest. The adsorption process of CO_2_ by the adsorbent is affected by both physical adsorption and chemical adsorption and CO_2_ adsorption is related to the adsorption sites of the adsorbent rather than a single monolayer adsorption.

(5) In the simulated flue gas, SO_2_ and HCl react with K_2_CO_3_ to produce new substances, which causes the adsorbent to gradually fail and K_2_CO_3_/AC has the best cycle failure performance.

## Figures and Tables

**Table 1:** Actual loading of different supports.

Theoretical Loading (%)	Actual Loading (%)				
	AC	Al_2_O_3_	5A	CFA	PFA
10	9.4	7.7	8.4	8.5	8.9
30	28.6	21.2	24.3	26.2	27.5
50	44.3	32.5	37.5	39.6	41.3

**Table 2:** Actual loading of different supports by XRF.

Theoretical Loading (%)	Actual Loading (%)				
	AC	Al_2_O_3_	5A	CFA	PFA
10	9.63	7.68	8.45	8.64	8.91
30	28.72	21.32	24.24	26.27	27.65
50	44.32	32.45	37.73	39.81	41.36

**Table 3:** Pore structure parameters of adsorbents before and after loading with the optimal theoretical loading.

Samples	BET Specific Surface Area m^2^·g^−1^	Cumulative Pore Volume cm^3^·g^−1^	Fractal Dimension	Pore Richness *Z*	Relative Pore Volume %	
					Micropore and Mesopore	Macropore
K_2_CO_3_	0.78	0.0073	2.0987	106.849	99.22	0.78
AC	493.87	0.3738	2.9050	1321.215	95.56	4.44
K_2_CO_3_/AC-30%	200.94	0.1630	2.9009	1232.761	96.84	3.16
Al_2_O_3_	18.54	0.0445	2.4512	416.629	93.65	6.35
K_2_CO_3_/Al_2_O_3_-30%	7.13	0.0275	2.5325	259.27	98.46	1.54
5A	44.75	0.1012	2.7958	443.061	92.61	7.39
K_2_CO_3_/5A-30%	8.53	0.0325	2.7789	262.403	96.00	4.00
CFA	80.85	0.0194	2.8775	4167.371	96.31	3.69
K_2_CO_3_/CFA-30%	47.02	0.0119	2.8543	3950.847	95.44	4.56
PFA	99.70	0.0257	2.8733	3879.494	93.81	6.19
K_2_CO_3_/PFA-30%	73.17	0.0199	2.8783	3676.935	93.35	6.65

**Table 4:** Fitting parameters of different sorbents.

Sorbents (30% Loading)	Pseudo-First Order Kinetic Equation			Pseudo-Second Order Kinetic Equation			Intra-Particle Diffusion Kinetic Equation			Elovich Equation		
	*R* ^2^	*k*_1_	*q_e_*	*R* ^2^	*k*_2_	*q_e_*	*R* ^2^	*k_id_*	*c*	R^2^	α	β
K_2_CO_3_/5A	0.9931	2.38 × 10^−4^	1379	0.9985	2.25 × 10^−9^	1793	0.9728	12.0765	−26	0.9973	0.3901	2.01 × 10^−3^
K_2_CO_3_/Al_2_O_3_	0.9975	3.31 × 10^−5^	1138	0.9956	1.75 × 10^−9^	1367	0.8789	8.4099	−208	0.9712	0.3784	3.15 × 10^−3^
K_2_CO_3_/CFA	0.9994	6.28 × 10^−4^	2429	0.9999	2.59 × 10^−7^	3605	0.9969	20.1509	−294	0.9995	0.5409	3.31 × 10^−4^
K_2_CO_3_/PFA	0.9998	7.09 × 10^−4^	5524	0.9999	5.12 × 10^−7^	8386	0.9962	32.3145	−727	0.9993	0.5851	7.92 × 10^−4^
K_2_CO_3_/AC	0.9995	7.39 × 10^−4^	6404	0.9999	6.46 × 10^−7^	10199	0.9948	36.4391	−957	0.9991	0.8879	2.54 × 10^−4^
